# Pharmacological Inhibition of Glutaminase 1 Attenuates Alkali-Induced Corneal Neovascularization by Modulating Macrophages

**DOI:** 10.1155/2022/1106313

**Published:** 2022-03-19

**Authors:** Yifan Feng, Xi Yang, Jinhai Huang, Minqian Shen, Liyang Wang, Xiuping Chen, Yuanzhi Yuan, Chunqiong Dong, Xiaoping Ma, Fei Yuan

**Affiliations:** ^1^Department of Ophthalmology, Zhongshan Hospital, Fudan University, Shanghai, China; ^2^Eye Institute and Department of Ophthalmology, Eye & ENT Hospital, Fudan University, Shanghai, China; ^3^NHC Key Laboratory of Myopia (Fudan University), Key Laboratory of Myopia, Chinese Academy of Medical Sciences, Shanghai, China

## Abstract

Corneal neovascularization (CoNV) in response to chemical burns is a leading cause of vision impairment. Although glutamine metabolism plays a crucial role in macrophage polarization, its regulatory effect on macrophages involved in chemical burn-induced corneal injury is not known. Here, we elucidated the connection between the reprogramming of glutamine metabolism in macrophages and the development of alkali burn-induced CoNV. Glutaminase 1 (GLS1) expression was upregulated in the mouse corneas damaged with alkali burns and was primarily located in F4/80-positive macrophages. Treatment with a selective oral GLS1 inhibitor, CB-839 (telaglenastat), significantly decreased the distribution of polarized M2 macrophages in the alkali-injured corneas and suppressed the development of CoNV. In vitro studies further demonstrated that glutamine deprivation or CB-839 treatment inhibited the proliferation, adhesion, and M2 polarization of bone marrow-derived macrophages (BMDMs) from C57BL/6J mice. CB-839 treatment markedly attenuated the secretion of proangiogenic factors, including vascular endothelial growth factor-A (VEGF-A) and platelet-derived growth factor-BB (PDGF-BB) from interleukin-4- (IL-4-) regulated M2 macrophages. Our findings revealed that GLS1 inhibition or glutamine deprivation prevented alkali-induced CoNV by inhibiting the infiltration and M2 polarization of macrophages. This work suggests that pharmacological GLS1 inhibition is a feasible and effective treatment strategy for chemical burn-related CoNV in humans.

## 1. Introduction

The cornea is a transparent tissue that plays important roles in vision and light refraction. The cornea contains antiangiogenic factors that maintain the avascular characteristics referred to as “angiogenic privilege” [1]. However, many pathological insults, such as infection, chemical burns, and trauma, trigger new pathological blood vessels to grow from the limbus toward the center of the cornea via a process known as corneal neovascularization (CoNV). Advanced CoNV results in corneal opacity and irreversible blindness [2]. Although the global impact is not known, the incidence rate of CoNV is estimated as 1.4 million per year in the United States, which presents a great challenge to ophthalmologists [3].

Macrophages play a crucial role in the modulation of CoNV via the secretion of various inflammatory and angiogenic mediators [4–7]. Polarized macrophages are broadly classified into M1 (classical activation) and M2 (alternative activation) subtypes based on function. M1 macrophages are activated by lipopolysaccharide (LPS), interferon-*γ* (IFN-*γ*), and Toll-like receptor (TLR) to produce proinflammatory cytokines and tissue inflammation. Conversely, M2 macrophages are stimulated by interleukin-4 (IL-4) and/or IL-13 (M2a subtype) or IL-10 (M2c subtype) and drive immune regulation and tissue remodeling [8, 9]. Notably, macrophages polarized toward the M2 phenotype have a higher angiogenic potential than M1 macrophages [10]. However, current knowledge of the regulation of macrophage phenotypes and functions, particularly during CoNV formation, is not complete.

The metabolic characteristics of M1 and M2 macrophages are different [11]. Extensive research demonstrated that metabolic remodeling in macrophage polarization played important roles in many inflammatory responses and diseases [12, 13]. Glutamine is the most abundant nonessential amino acid in the circulation, and it has multiple metabolic uses in cells. Glutamine may be initially converted into glutamate then further metabolized to *α*-ketoglutarate (*α*-KG), which is a key intermediate in the trichloroacetic acid (TCA) cycle. GLS is a required enzyme in the first step of the glutaminolysis (conversion of glutamine to *α*-KG) pathway. There are two distinct but related GLS genes in mammals, *GLS1* (the kidney isoform) and *GLS2* (the liver isoform) [14]. GLS1 is expressed ubiquitously, but GLS2 is expressed primarily in the liver. Glutaminolysis is an important metabolic factor controlling macrophage reprogramming and phenotypic polarization [15]. Several studies revealed that *α*-KG derived from glutaminolysis was essential for M2 polarization [16–18]. Therefore, we hypothesized that GLS1 is a potential target in macrophages for the prevention and treatment of CoNV.

We demonstrated that GLS1 expression was upregulated in the murine corneas damaged by alkali burn injury and extensively located in macrophages. Treatment with a selective oral GLS1 inhibitor, CB-839 (telaglenastat), significantly decreased the distribution of Mrc1-positive M2 macrophages and suppressed CoNV after alkali burn injury. In vitro studies further demonstrated that glutamine deprivation or CB-839 treatment inhibited the proliferation, adhesion, and IL-4-induced secretion of proangiogenic factors, including vascular endothelial growth factor-A (VEGF-A) and platelet-derived growth factor-B (PDGF-B), in macrophages. Taken together, our study revealed the therapeutic potential of pharmacological inhibition of GLS1 in macrophages as an attractive avenue to suppress the progression of CoNV disorders.

## 2. Materials and Methods

### 2.1. Animals and Ethics Statement

C57BL/6J mice aged 6-8 weeks were purchased from SLAC Laboratory Animal Co., Ltd. (Shanghai, China). All studies complied with the ARVO Statement for the Use of Animals in Ophthalmic and Vision Research, and all procedures were approved and monitored by the Institutional Animal Care and Use Committee of Zhongshan Hospital, Fudan University (No. 2019-285).

### 2.2. CoNV Model Establishment and Treatment

CoNV was induced using a previously described alkali burn method [19, 20]. Briefly, mice were anesthetized with an injection of 1% sodium pentobarbital (Sigma-Aldrich, St. Louis, MO, USA), and a 3.0 mm diameter circular piece of NaOH-soaked (1 M; Sigma; CAT No. S8045) filter paper was placed on the surface of the right cornea for 30 s. After alkali exposure, the ocular surface was rinsed with a sterile saline solution for 1 min. After the alkali burn, the animals were treated with vehicle (1% DMSO; Sigma; CAT No. D2650) or CB-839 (40 mg/kg; Selleck; CAT No. S7655) via intraperitoneal injection once daily, according to a previous study [21]. The experiment was terminated using euthanasia 3 and 7 days after the alkali burn, and all eyes were enucleated for subsequent analyses.

### 2.3. Evaluation of CoNV

Seven days after the alkali burns, the mouse corneas were examined and photographed using a digital camera attached to a slit lamp microscope (Zeiss, Jena, Germany). CoNV was observed, and clinical assessments were performed according to an existing standard [19]: opacity (scale 0-3), NV score (scale 0-3), and vessel size (scale 0-3). Two independent observers scored the corneas, and the final score was the average of the two scores.

### 2.4. Histological Staining

The corneas were harvested at the end of the experiment, cut into small pieces, and fixed in 4% paraformaldehyde (PFA). The samples were processed using routine dehydration in an ethanol gradient, cleared with xylene, and embedded in paraffin. The coronal section was cut into 5 *μ*m thick tissue sections and mounted on slides. The slides were deparaffinized, stained with hematoxylin and eosin (H&E), dehydrated with alcohol, and mounted in neutral balsam using an Eclipse 50i clinical microscope (Nikon Corporation, Tokyo, Japan). Most sections were taken from the central region of the cornea, and CoNV was evaluated in at least two sections from each eye.

### 2.5. Cell Culture

Bone marrow-derived macrophages (BMDMs) were collected as described previously [22]. Briefly, bone marrow cells were flushed from the tibiae and femurs of C57BL/6 mice and seeded at 1 × 10^6^ cells/well in 24-well culture plates in DMEM supplemented with 10% FBS, L-glutamine (2 mM), 1% penicillin/streptomycin, and 15% L929-conditioned medium (LCM). Cells were incubated at 37°C in a humidified 5% CO_2_ incubator. On day 7, adherent macrophages were gently scraped, and all the cells were resuspended, centrifuged, and seeded at 2 × 10^6^ cells/well in 6-well culture plates in culture medium for 24 h. The purity of the BMDMs was routinely >95%, as confirmed by integrin alpha M (ITGAM; CD11b) staining (Cell Signaling Technology; CAT No. 17800). For glutamine deprivation, cells were cultured in glutamine-free and pyruvate-free DMEM with 4.5 g/l glucose (Thermo Fisher Scientific; CAT No. SH30081) and 10% dialyzed FBS (Thermo Fisher Scientific; CAT No. 30067334).

Mouse retinal microvascular endothelial cells (MRMECs) were purchased from Cell Biologics (CAT No. C57-6065). The cells were cultured in complete mouse endothelial cell medium (ECM) mixed with 1% penicillin and streptomycin antibiotics (Cell Biologics; CAT No. M1168) under 5% CO_2_ at 37°C. Cells from passages 3 to 7 were used for experiments.

### 2.6. Glutamine Consumption Assays

Glutamine levels were determined using a Glutamine Detection Assay Kit (BioVision; CAT No. K556-100) in accordance with the manufacturer's instructions. BMDMs were cultured in DMEM containing 2 mM glutamine and treated with vehicle or CB-839 (1 nM to 5 *μ*M) for 6 h, and the cell supernatant was harvested. Glutamine consumption was calculated by subtracting the detected concentration of glutamine in the medium from the original glutamine concentration and expressed as the fold change. All values were normalized to protein concentrations.

### 2.7. [^3^H]-Thymidine Incorporation

[^3^H]-Thymidine incorporation was measured to determine the effect of CB-839 on BMDM proliferation. Cells were treated with vehicle or CB-839 (1 nM to 5 *μ*M) for 24 h, and 1 *μ*Ci/ml [^3^H]-thymidine (PerkinElmer; CAT No. NET027E250UC) was added. After incubation for 24 h, cells were fixed with 100% ethanol for 15 min at 4°C, precipitated with cold 10% TCA, and lysed with 0.1 N NaOH. The amount of [^3^H]-thymidine incorporated into DNA was measured using liquid scintillation counting (Beckman LS 7500, Fullerton, CA) and corrected for cell number.

### 2.8. Flow Cytometry Analysis of Apoptosis

Apoptosis was detected using the Annexin V-FITC kit (KeyGen Biotech; CAT No. KGA108) as described previously [23]. Briefly, BMDMs were treated with vehicle or CB-839 (1 nM to 1 *μ*M) for 24 h, harvested, washed, and incubated with Annexin V-FITC and propidium iodide for 15 min at room temperature in the dark. Cells were immediately analyzed using a BD FACSCalibur flow cytometry system (BD Biosciences, San Jose, CA, USA).

### 2.9. Cell Adhesion Assays

Cell adhesion assays were performed as described in a previous study [24]. MRMECs were grown to confluence in 6-well plates at 37°C in a humidified 5% CO_2_ incubator. BMDMs were labeled with 5 *μ*mol/l calcein-acetoxymethyl ester (calcein/AM, Thermo Fisher Scientific; CAT No. C1429) and cultured in an incubator for 30 min. The labeled cells were washed twice with PBS and resuspended in serum-free medium. The culture medium of MRMECs was removed, and the resuspensions of labeled BMDMs were added onto monolayers of MRMECs. After 1 h incubation, the plates were rinsed twice with medium without serum. The adherent BMDMs on MRMECs were counted using a fluorescence microscope (Olympus IX71, Tokyo, Japan). Three fields were captured per experimental condition. Individual treatments were performed in duplicate, and the entire set of experiments was repeated three times.

### 2.10. Macrophage Polarization

Differentiated BMDMs were harvested, counted, and suspended in DMEM (without LCM) overnight and pretreated with vehicle or CB-839 (100 nM) for 24 h. BMDMs were stimulated with 10 ng/ml LPS (Sigma; CAT No. L4391) for M1 activation and 20 ng/ml interleukin-4 (IL-4) (PeproTech; CAT No. 214-14) for M2 activation.

### 2.11. Metabolic Assays

BMDMs were seeded at 1 × 10^6^/ml (replicates of three) and treated with vehicle or CB-839 (100 nM) for 24 h, followed by incubation in regular DMEM (for unlabeled samples) or glutamine-free DMEM supplemented with ^13^C_5_,^15^N_2_-labeled L-glutamine (Cambridge Isotope Laboratories; CAT No. CNLM-1275-H) for 18 h in the presence of vehicle or drug. After washing with ice-cold PBS, metabolites were extracted from cells in 0.5 ml lysis buffer containing methanol/acetonitrile/water (2 : 2 : 1). Samples were centrifuged at 16,000 × *g* for 15 min at 4°C, and the supernatants were collected for analyses using ultrahigh-performance liquid chromatography and mass spectrometry (LC-MS) as described previously [25].

### 2.12. Seahorse Oxygen Consumption Rates

The oxygen consumption rate (OCR) is widely used as a proxy for mitochondrial oxidative phosphorylation (OXPHOS). OCR measurements were performed using a Seahorse Bioscience XF-96 instrument as previously described [18]. The XF96 protocol consisted of basal OCR (1 measurement/1.5 min), injection of 1.0 mM oligomycin (Sigma; CAT No. 75351), injection of 0.5 mM FCCP (Sigma; CAT No. C2920) with four measurements of uncoupled OCR (1 measurement/1.5 min), and a final injection of 1 mM rotenone (Sigma; CAT No. R8875) and antimycin A (1 mM; Sigma; CAT No. A8674). BMDMs were plated at a density of 1 × 10^5^ cells per well in 100 *μ*l XF running buffer in 96-well plates. Cells treated with CB-839 were pretreated with 1 *μ*M drug for 12 h.

### 2.13. Conditioned Media Preparation

The culture supernatant of BMDMs containing a variety of growth factors and cytokines was collected and used in tube formation and choroid sprouting assays as described below. Briefly, BMDMs were activated with IL-4 (40 ng/ml) for 24 h with vehicle or CB-839 pretreatment, collected by centrifugation at 500 rpm for 5 min, and washed with PBS (pH 7.4) to remove the IL-4. Polarized BMDMs were incubated in serum-free medium for 24 h, and culture supernatants were collected as conditioned medium (BMDM-CM). The CM was centrifuged at 4,000 rpm for 5 min to remove debris and stored at -80°C.

### 2.14. Tube Formation Assay

The tube formation assay was performed as previously described. Briefly, aliquots (150 *μ*l) of growth factor-reduced Matrigel (BD Biosciences; CAT No. BD354230) were added to a prechilled 48-well plate and incubated at 37°C for 30 min. The MRMECs were resuspended in ECM alone or ECM containing 50% CM (ECM : BMDM − CM = 1 : 1) and seeded onto the gel (2 × 10^4^ cells/well). Five random fields from each well were chosen and photographed using inverted microscopy (Olympus) after 8 hours. Networks of tube-like structures were measured using ImageJ software (National Institutes of Health).

### 2.15. Ex Vivo Choroid Sprouting Assay

Ex vivo choroid sprouting assays were performed as previously described [26, 27]. Briefly, the choroid RPE complex (“choroid explants”) was dissected from the mouse eyes, and the peripheral area of the complexes was cut into approximately 1 × 1 mm pieces. The explants were immediately embedded in 30 *μ*l growth factor-reduced Matrigel (BD Biosciences) in 24-well tissue culture plates. The choroidal explants were grown in ECM or ECM/BMDM-CM at 37°C with 5% CO_2_. After 3 days of ex vivo culture, images of the choroid sprouting were recorded, and the sprouting area was quantified using ImageJ software.

### 2.16. Real-Time Reverse Transcriptase PCR (RT-PCR)

Total RNA from BMDMs and the corneas was isolated using TRIzol (Thermo Fisher Scientific), and 500 ng total RNA was used for cDNA synthesis with PrimeScript RT Master Mix (Takara; CAT No. RR036A). RT-PCR was performed using an ABI PRISM 7500 Fast Real-Time PCR System (Applied Biosystems, Foster City, CA, USA) and TB Green® Premix Ex Taq™ (Takara; CAT No. RR420A) according to the manufacturer's instructions. The sequences of specific primers are presented in Supplementary Table S1. Beta-actin was used as an internal control for mRNA assays. The gene expression levels were calculated using the 2^-*ΔΔ*Ct^ method after normalization to the endogenous reference [23].

### 2.17. Western Blotting

Protein extracts were isolated from the mouse corneas using RIPA lysis buffer (Beyotime Biotechnology; CAT No. P0013B) containing a protease and phosphatase inhibitor cocktail (Beyotime; CAT No. P1046). Following centrifugation of the lysates at 12,000 × *g* for 15 min at 4°C, the supernatants were collected, and total protein was quantified using a BCA assay kit (Beyotime; CAT No. P0012). Equal amounts of protein from each sample were separated using 10% SDS-PAGE and transferred onto polyvinylidene fluoride membranes (Millipore; CAT No. IPFL00010). Membranes were blocked with 5% skimmed milk (*w*/*v*) in TBS-0.05% Tween-20 (TBST) buffer (Sangon Biotech; CAT No. C520009) at room temperature for 2 h then incubated with primary antibodies at 4°C overnight. Following primary antibody incubation, the membranes were incubated with secondary antibodies for 1 h. The reactive bands were detected and observed using an enhanced chemiluminescence (ECL) kit (Thermo Fisher Scientific; CAT No. 32106). Densitometric quantifications of bands were performed with ImageJ software (National Institutes of Health) using *β*-actin as an internal reference. The antibodies for Western blot analyses are listed in Supplementary Table S2.

### 2.18. Immunofluorescent Staining

BMDMs with different treatments cultured on coverslips were washed and fixed with 4% paraformaldehyde for 20 min and extracted with a 0.5% Triton X-100 solution (Beyotime Biotechnology; CAT No. P0096) for 5 minutes. After blocking with TBST containing 5% normal goat serum (Sigma; CAT No. G9023), cells were incubated with rabbit anti-PIGF (1 : 100; Abcam; CAT No. ab9572) and rabbit anti-PDGFB (1 : 100 dilution; Abcam; CAT No. ab23914) primary antibodies for 1 h. Cells were washed and incubated with Alexa Fluor 594-conjugated goat anti-rabbit secondary antibody (Abcam; CAT No. ab150080) for 1 h, followed by DAPI (Beyotime; CAT No. C1002) for 3 min. Images were captured using an inverted microscope (Olympus).

Corneal sections were incubated in 5% normal goat serum in TBST/0.5% Triton X-100 for 1 h to block nonspecific binding of the primary antibody. Sections were incubated with mouse anti-GLS1 (1 : 100 dilution; Proteintech; CAT No. 66265-1-Ig), rabbit anti-F4/80 (1 : 400 dilution; Cell Signaling Technology; CAT No. 70076), and rabbit anti-CD31 (1 : 100 dilution; Abcam; CAT No. ab222783) overnight at 4°C. After 3 washes in PBS, sections were incubated for 1 h with a 1 : 200 dilution of Alexa Fluor 488-conjugated goat anti-mouse secondary antibody (Abcam; CAT No. ab150113) or Alexa Fluor 594-conjugated goat anti-rabbit secondary antibody (Abcam; CAT No. ab150080). Sections were rinsed in PBS and incubated with DAPI for 15 min. Images were captured using an inverted microscope (Olympus).

### 2.19. Enzyme-Linked Immunosorbent Assay (ELISA)

The concentrations of VAGF-A (CAT No. MMV00), PDGF-BB (CAT No. MBB00), IL-10 (CAT No. M1000B), CCL22 (CAT No. M2200), IL-1*β* (CAT No. MLB00C), and TNF-*α* (CAT No. MTA00B) in BMDM-CM were measured using Quantikine ELISA kits (R&D Systems) according to the manufacturer's protocols. The concentrations of these proteins were calculated from a standard curve.

### 2.20. Statistical Analysis

Results are presented as the means ± SEM. Parametric statistical analysis between two groups was evaluated using two-tailed Student's *t*-tests. The Mann-Whitney test was used for nonparametric statistical analyses between two groups. One-way analysis of variance (ANOVA) followed by Tukey's or Dunnett's post hoc test was used for statistical comparisons of gene expression. *P* < 0.05 was considered statistically significant. These tests were performed using GraphPad Prism 7 software (GraphPad Inc., La Jolla, CA, USA).

## 3. Results

### 3.1. Targeting Glutaminolysis Changes the Basic Biology of Macrophages In Vitro

**Figure 1:**
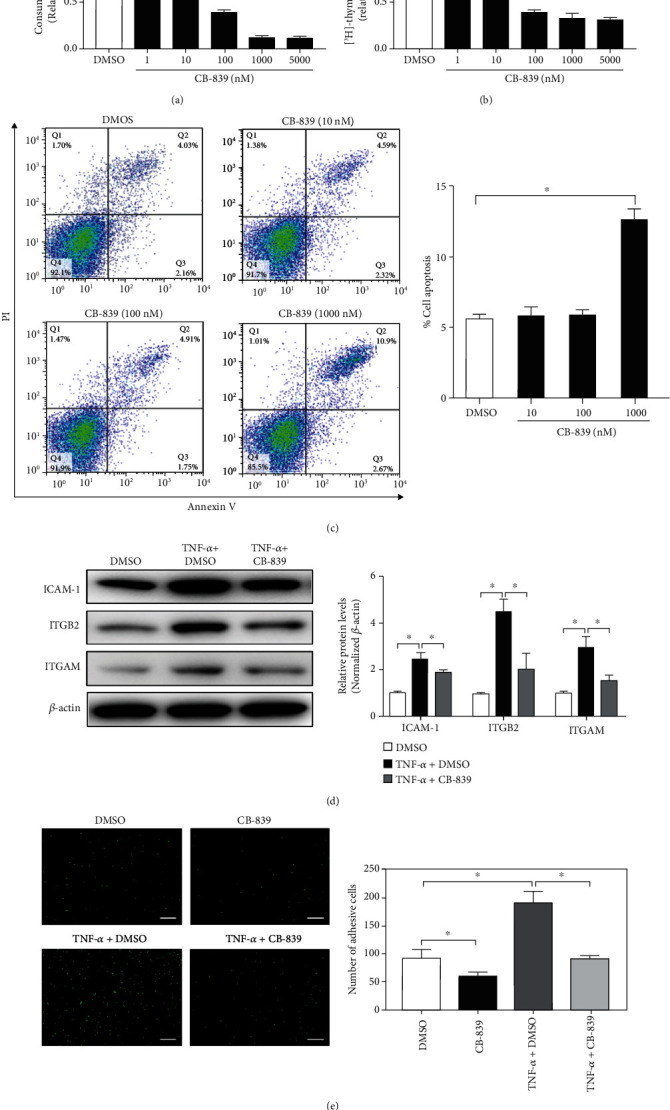
Targeting glutaminase 1 (GLS1) changes the basic biology of macrophages in vitro. (a) Glutamine consumption rates for BMDMs measured after DMSO or different concentrations of CB-839 treatment. Medium was collected after 6 hours of treatment and analyzed for glutamine. (b) [^3^H]-Thymidine incorporation in DNA in BMDMs treated with different doses of CB-839 for 24 h. (c) The percentage of apoptotic BMDMs 24 h after treatment with DMSO or different concentrations of CB-839 was determined by Annexin-V/PI staining and flow cytometry. (d) Representative Western blot and quantitative analysis of ITGAM, ITGB2, and ICAM-1 protein expression in BMDMs treated with CB-839 (1 *μ*M) for 24 h. *β*-Actin served as an endogenous reference for normalization. (e) After stimulating BMDMs with TNF-*α* (10 ng/ml) alone or combined with CB-839 (1 *μ*M) treatment for 1 h, fluorescence microscope was applied to observe the number of adhesion of calcein-AM-labeled BMDMs to monolayers of MRMECs. Each experiment repeated three times, and data in graphs represent the means ± SEM. ^∗^*P* < 0.05.

To determine the importance of glutamine metabolism on macrophages, we cultured BMDMs in the presence or absence of glutamine. Notably, glutamine deprivation significantly inhibited proliferation and adhesion and promoted apoptosis of BMDMs (Supplementary Figure S1A–C). Glutamine withdrawal depleted the intracellular pool of TCA cycle metabolites (Supplementary Figure S1D). We treated BMDMs with the GLS1-specific inhibitor CB-839 (Supplementary Figure S2A) [28] and observed apparent inhibitory effects on glutamine consumption in BMDMs (Figure 1(a)). CB-839 treatment dose-dependently reduced cell proliferation (Figure 1(b)) and induced apoptosis of BMDMs (Figure 1(c)). We further assessed the expression of the adhesion molecules ITGAM, integrin beta 2 (ITGB2), and intercellular adhesion molecule-1 (ICAM-1) upon TNF-*α* stimulation to examine whether glutamine metabolism affected the activation of macrophages under conditions of inflammation. GLS1 inhibition altered the expression of these markers (Figure 1(d)) and the adhesion of BMDMs (Figure 1(e)). CB-839 notably reduced the concentration of a number of key metabolite downstream of glutamate (Supplementary Figure S2B) and the rate of oxygen consumption (Supplementary Figure S2C). Supplementation with exogenous glutamate restored the biological and metabolic properties of CB-839-treated BMDMs (Supplementary Figure S2B-D). Taken together, these observations demonstrated that targeting glutaminolysis or GLS1 changed the basic biology of macrophages.

### 3.2. Targeting Glutaminolysis Modulates Macrophage Polarization In Vitro

**Figure 2:**
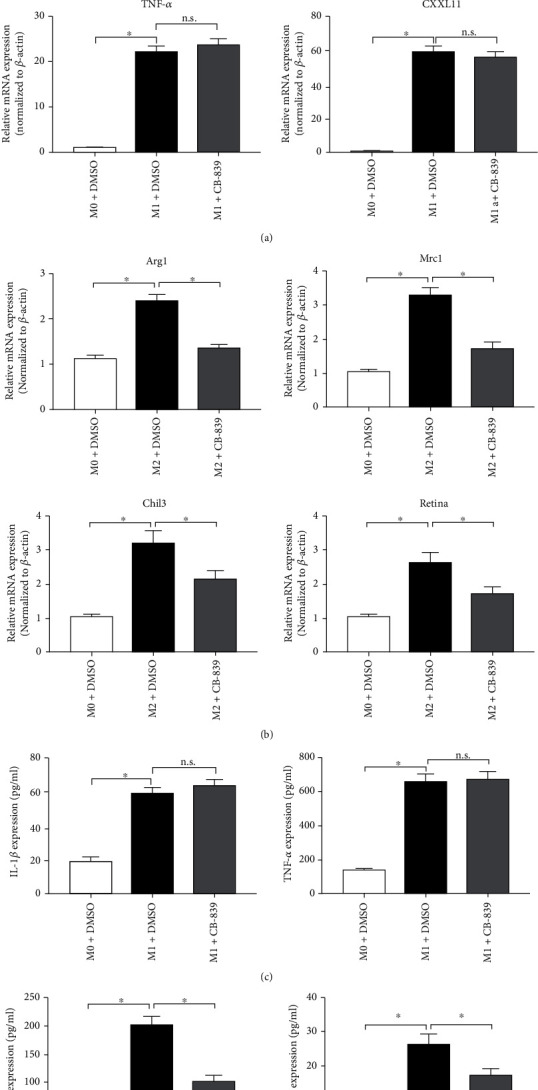
Targeting glutaminase 1 (GLS1) activity modulates polarization of macrophages in vitro. RT-PCR analysis of M1 marker genes (a) and M2 marker genes (b) in uncommitted (M0) or LPS (M1) or IL-4 (M2)-treated BMDMs under various culture conditions for 12 h. ELISA assay was performed to examine the concentration of M1 markers (c) and M2 markers (d) in culture medium of BMDMs under various culture conditions for 24 h. Each experiment repeated three times, and data in graphs represent the means ± SEM. ^∗^*P* < 0.05.

To investigate how glutaminolysis regulated macrophage polarization, we determined the effects of GLS1 inhibition and glutamine deprivation in BMDMs treated with IL-4 or LPS. As shown in Figures 2(a) and 2(c), treatment with CB-839 impaired the expression of M2-specific marker genes (arginase 1 [Arg1], chitinase-like 3 [Chil3], resistin-like alpha [Retnla], and mannose receptor C-type 1 [Mrc1]) and cytokine production (IL-10 and C-C motif chemokine 22 [CCL22]) in IL-4-induced BMDMs. In contrast, CB-839 treatment did not alter the high expression of M1-specific marker genes or cytokines (IL-1*β*, TNF-*α*, C-X-C motif chemokine ligand 11 [CXCL11], and IL-6) in BMDMs stimulated with LPS (Figures 2(b) and 2(d)). The effects of targeting glutaminolysis in M2 macrophages were also confirmed using glutamine deprivation (Supplementary Figure S3A). Notably, deprivation of glutamine boosted the expression of IL-1*β*, IL-6, and TNF-*α* in LPS-stimulated BMDMs, which was different from the effect of CB-839 treatment (Supplementary Figure S3B). Together, these results indicate that glutaminolysis is crucial for supporting an M2 phenotype in macrophages.

### 3.3. Targeting Glutaminolysis Suppresses Macrophage-Induced Angiogenesis In Vitro

**Figure 3:**
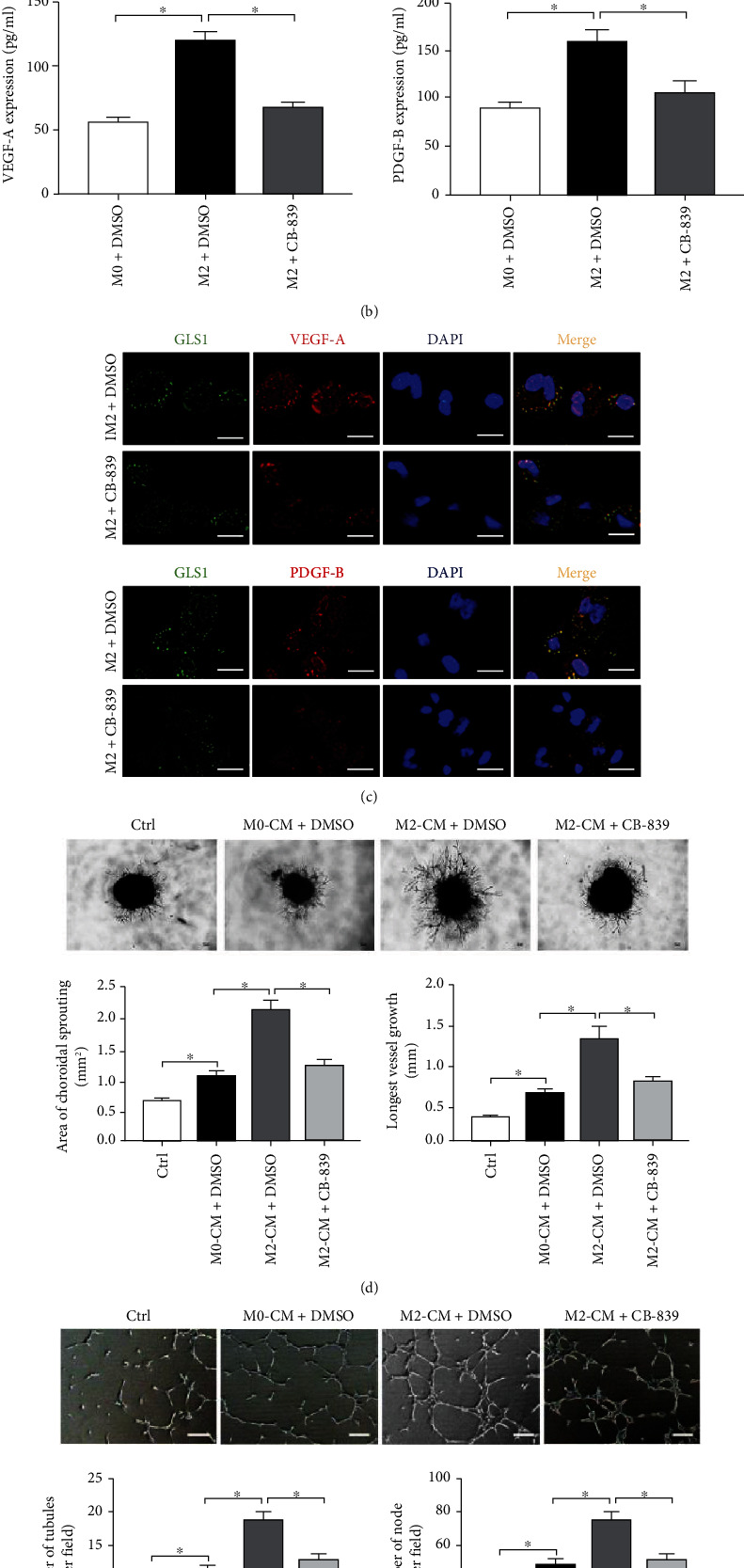
Targeting glutaminase 1 (GLS1) suppresses macrophage-mediated angiogenesis in vitro. RT-PCR and ELISA assays were performed to determine the mRNA (a) and protein (b) levels of VEGF-A and PDGF-BB in uncommitted (M0) or IL-4 (M2)-treated BMDMs under various culture conditions for 12 and 24 h, respectively. (c) Immunofluorescence double staining of GLS1 (green) and VEGF-A or PDGF-BB (green) in M2 phenotypic BMDMs treated with or without CB-839 treatment. (d) Choroidal explants isolated from C57BL/6J mice were treated with conditioned media (CM) from the M0 or M2 phenotypic BMDMs alone or M2 BMDMs plus CB-839 (1 *μ*M) treatment, and the angiogenic potency of choroidal explants was assessed using a Matrigel-based choroidal sprouting assay. Sprouting area and maximal extension of angiogenesis from the choroidal tissue edge were measured by ImageJ (*n* = 6/group). Scale bar = 100 *μ*m. (e) MRMECs were treated with CM from BMDMs described in (d) for 6 h, and the tube formation of MRMECs was assessed using a Matrigel assay. The number of tubules and nodes formed was quantified and analyzed by ImageJ (*n* = 4/group, five fields were calculated per sample). Scale bar = 50 *μ*m. Each experiment repeated three times, and data in graphs represent the means ± SEM. ^∗^*P* < 0.05.

Previous studies showed that macrophages polarized toward an M2 phenotype had high angiogenic potential via the release of a number of proangiogenic factors [10]. Therefore, we examined whether CB-839 affected proangiogenic factor secretion from IL-4-stimulated macrophages. RT-PCR demonstrated that IL-4 stimulation promoted the mRNA expression of proangiogenic factors, including VEGF-A, PDGF-B, insulin-like growth factor-1 (IGF-1), placental growth factor (PLGF), and fibroblast growth factor-2 (FGF-2), and pretreatment with CB-839 significantly attenuated the mRNA levels of VEGF-A and PDGF-B (Figure 3(a); Supplementary Figure S4). Notably, the CB-839-mediated inhibition of VEGF-A and PDGF-BB protein expression in IL-4-stimulated macrophages was further confirmed using ELISA and immunofluorescence staining (Figures 3(b) and 3(c)). To identify whether CB-839 reversed M2 macrophage-induced angiogenesis, we performed choroid sprouting assays and MRMEC tube formation assays with BMDM-CM. As shown in Figures 3(d) and 3(e), CM from IL4-treated BMDMs (M2-CM+DMSO group) obviously increased choroidal vessel outgrowth and luminal formation compared to the control or DMSO-treated M0-CM groups, and CM from CB-839-pretreated IL-4-stimulated BMDMs (M2-CM+CB-839 group) markedly inhibited choroidal angiogenesis and MRMEC tube formation. Collectively, these results support the hypothesis that inhibition of glutaminolysis affects the angiogenesis-promoting feature of M2 macrophages.

### 3.4. Expression of GLS1 Was Increased in the Mouse Corneas following Alkali Burn Injury

**Figure 4:**
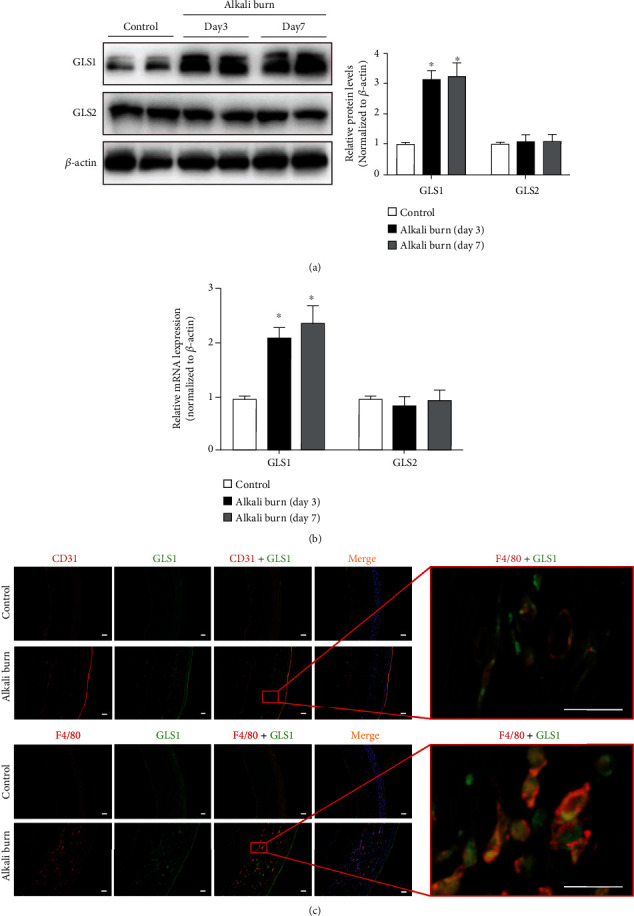
Expression of glutaminase 1 (GLS1) was increased in the mouse corneas following alkali burn injury. (a) Representative Western blot and quantitative analysis of GLS1 and GLS2 protein expression in the mouse corneas without and with alkali burn. *β*-Actin served as an endogenous reference for normalization. (b) Immunofluorescence double staining of GLS1 (green) and endothelium marker CD31 (red) or macrophage marker F4/80 (red) in the corneas without and with alkali burn on day 7. Scale bars: 100 *μ*m. *n* = 6 − 8/group for Western blotting and *n* = 4 − 6/group for immunofluorescence. Each experiment repeated three times, and data in graphs represent the means ± SEM. ^∗^*P* < 0.05, compared with the control group.

To further characterize the potential impact of the glutaminolysis-dependent effect of macrophages on CoNV development in vivo, we further examined the expression of GLS1 and GLS2 in a mouse model of alkali burn-induced CoNV. GLS1 and GLS2 mRNA and protein were detectable in the untreated corneas and the alkali-injured corneas, but only GLS1 expression was markedly increased 3 and 7 days after alkali injury (Figures 4(a) and 4(b)). To investigate which cells expressed GLS1 in the alkali-injured corneas, we performed double immunofluorescent staining for GLS1 and CD31 (endothelial cell marker) or F4/80 (differentiated macrophage marker). As shown in Figure 4(c), GLS1 expression was increased in the corneas of alkali-burned mice on day 7. Notably, GLS1 evidently colocalized with F4/80-positive macrophages but with few CD31-positive endothelial cells in the alkali-injured corneas.

### 3.5. Targeting GLS1 Inhibits M2 Polarization of Macrophages in the Alkali-Injured Mouse Cornea

**Figure 5:**
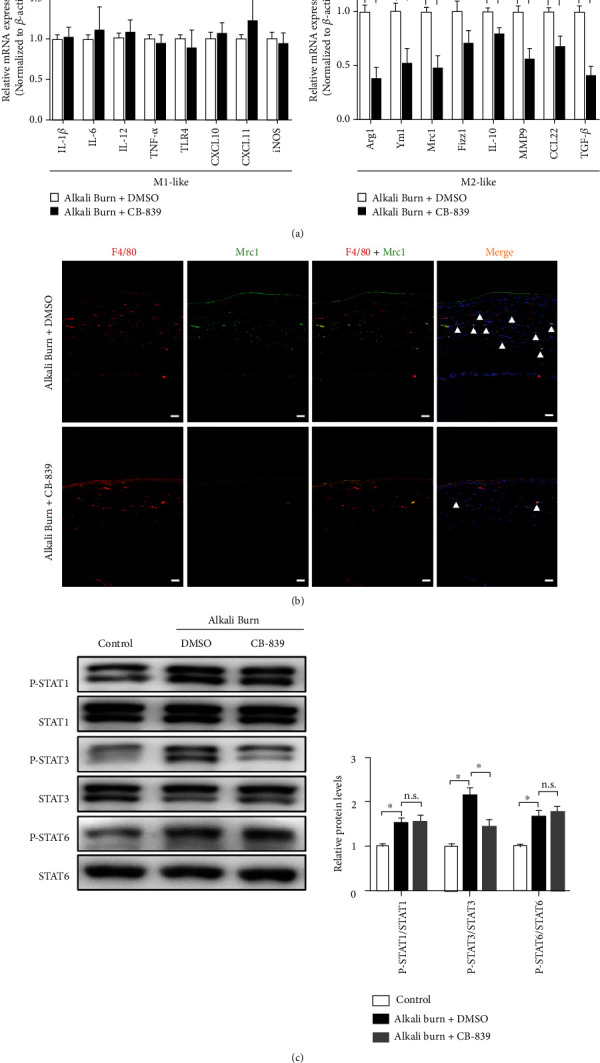
Targeting glutaminase 1 (GLS1) inhibits M2 polarization of macrophages in the alkali-injured mouse corneas. (a) RT-PCR analysis of M1 and M2 marker genes in the alkali-injured mouse corneas treated with DMSO or CB-839 (200 mg/kg) for 7 days. (b) Immunofluorescence double staining of F4/80 (red) and M2-specific marker Mrc1 (green) in the alkali-injured mouse corneas treated with DMSO or CB-839 for 7 days. Scale bars: 50 *μ*m. (c) Representative Western blot and quantitative analysis of STAT1, phospho-STAT1 (Tyr701), STAT3, phospho-STAT3 (Tyr705), STAT6, and phospho-STAT6 (Tyr641) in the normal corneas (control) and the alkali-injured corneas treated with DMSO or CB-839 for 7 days. *β*-Actin served as an endogenous reference for normalization. *n* = 6 − 8/group for RT-PCR or Western blotting and *n* = 4 − 6/group for immunofluorescence. Each experiment repeated three times, and data in graphs represent the means ± SEM. ns: no significance. ^∗^*P* < 0.05.

Glutaminolysis is critically involved in macrophage polarization. Therefore, we investigated the effects of CB-839 inhibition on macrophage polarization in the alkali-injured corneas. We observed lower mRNA levels of markers for M2-like macrophages (Arg-1, Chil3, Retnla, Mrc1, IL-10, matrix metalloproteinase 9 [MMP9], CCL22, and TGF-*β*) in the CB-839 group compared to the DMSO group and no significant difference in the mRNA levels of markers for M1-like macrophages (IL-1*β*, IL-6, IL-12, TNF-*α*, TLR4, CXCL10, CXCL11, and inducible nitric oxide synthase [iNOS]) (Figure 5(a)). Immunofluorescent staining revealed that the number of F4/80^+^/Mrc1^+^ double-labeled cells was notably increased in the injured corneas on 7 days after corneal alkali burn and was significantly decreased with GLS1 inhibition (Figure 5(b)). Signal transducer and activator of transcription 3 (STAT3) and STAT6 are well-known transcription factors that induce M2 macrophage activation, and STAT1 is a key transcription factor for M2 macrophages [8, 29]. Therefore, the phosphorylation levels of signal transducer and activator of transcription 1 (STAT1), STAT3, and STAT6 were detected using Western blot, and the results demonstrated an upregulation of phosphorylation in the alkali-injured corneas compared to the normal control corneas, but CB-839 treatment only suppressed the level of STAT3 phosphorylation (Figure 5(c)). These observations suggest that GLS1 activity is important for M2 macrophage polarization in the mouse corneas after alkali injury.

### 3.6. Targeting GLS1 Inhibits Alkali Burn-Induced CoNV in the Mouse Corneas

**Figure 6:**
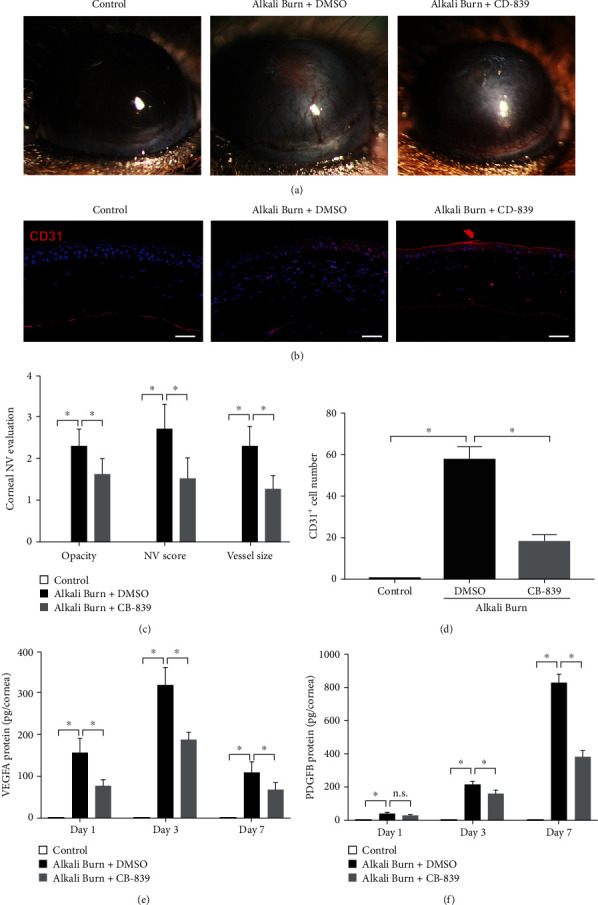
Targeting GLS1 inhibits alkali burn-induced neovascularization in the mouse corneas. (a) Representative images of the macroscopic CoNV appearance after DMSO or CB-839 treatment on day 7 after alkali burn injury. (b) Immunofluorescence staining of CD31 (red) in the mouse corneas from different groups. Scale bars: 100 *μ*m. (c) Statistical analysis of the clinical assessments for CoNV. (d) Statistical analysis of CD31-positive cell number. ELISA assay was performed to examine the concentration of VEGF-A (e) and PDGF-BB (f) in the normal corneas (control) and the alkali-injured corneas treated with DMSO or CB-839 (200 mg/kg) for 1, 3, and 7 days. *n* = 5 − 6/group for ELISA or immunofluorescence. Each experiment repeated three times, and data in graphs represent the means ± SEM. ^∗^*P* < 0.05.

**Figure 7:**
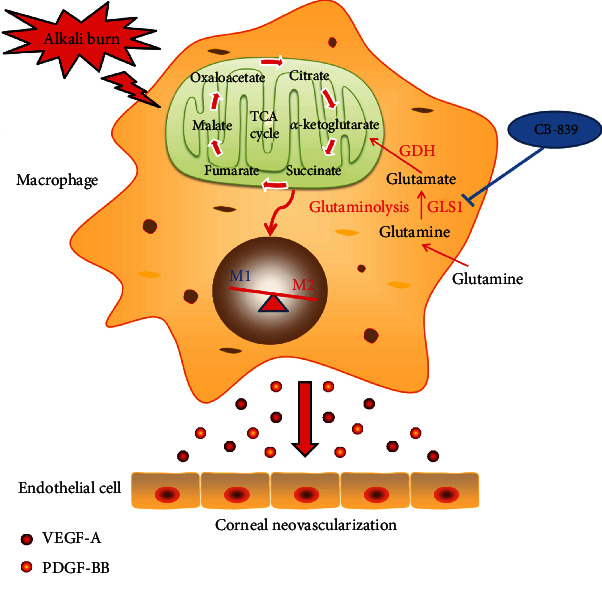
Schematic diagram showing that inhibited GLS1-mediated glutaminolysis attenuates alkali-induced corneal neovascularization by modulating polarization of macrophages.

We used the alkali burn-induced CoNV model to investigate whether CB-839 treatment affected neovascularization in vivo. Based on the morphological results of the cornea using the slit lamp and immunofluorescence staining for CD31, representative images were captured 7 days after alkali burn. The results showed that the alkali-burned corneas had extensive pathological vessel growth, as shown in Figures 6(a) and 6(b). The inhibition of GLS1 via intraperitoneal injection of CB-839 alleviated alkali burn-induced CoNV, which was demonstrated by reductions in the corneal opacity and area of CoNV (Figure 6(c)) and the number of CD31-positive cells (Figure 6(d)). Hematoxylin and eosin (H&E) staining was also performed to detect CoNV after alkali burn injury (Supplementary Figure S5A). CB-839 injection largely inhibited alkali-induced CoNV because the thickness of the corneas was significantly reduced (Supplementary Figure S5B). Notably, the number of new vessels was significantly increased in the mouse corneas 7 days after alkali burn, which was also largely attenuated by CB-839 treatment (Supplementary Figure S5C). Consistently, the ELISA results revealed that the VEGF-A and PDGF-B protein levels were elevated after alkali injury in DMSO-treated mice and decreased in CB-839-treated mice (Figures 6(e) and 6(f)), which suggests that blocking of GLS1 activity using CB-839 shows promise as a CoNV therapeutic (Figure 7).

## 4. Discussion

Chemical burns remain an important cause of corneal damage and potentially lead to visual impairment or loss due to tissue destruction and neovascularization formation [30, 31]. The inflammatory cell response plays a crucial role in chemical burn-induced corneal damage. Comprehensive observations of murine mode of corneal alkali burn reveal that inflammatory cells, such as neutrophils and macrophages, are activated and infiltrate into the damaged tissue, which peaks 1 and 7 days after injury, respectively [32, 33]. These cells subsequently produce a large number of proinflammatory cytokines and proangiogenic factors that induce multiple cellular signaling cascades and promote endothelial cell proliferation and new blood vessel formation. Therefore, therapeutic studies targeting inflammation and neovascularization in corneal burns have become more prevalent in recent years [34]. Alkali-induced CoNV is evident until 7 days [35]. Therefore, we focused on macrophages in the current study because these cells are a major source of proangiogenic molecules that are critical for the development of neovascularization.

Macrophages are a group of phenotypic heterogenic cells that are classified into two major subsets, classically activated (M1) and alternatively activated (M2), depending on the stimuli [8]. During development and tissue healing or regeneration, M2-polarized macrophages stimulate angiogenesis and facilitate tissue remodeling by secreting a number of proteases and growth factors [36, 37]. An increasing body of evidence revealed that the recruitment and M2 polarization of macrophages are closely associated with multiple models of ocular neovascularization, such as oxygen-induced retinopathy (OIR) [38, 39] and choroidal neovascularization (CNV) [4, 40, 41]. However, the specific roles of M2 macrophages in CoNV are not defined. The present study demonstrated that the number of Mrc1-positive M2 macrophages increased in the corneas after alkali burn injury. CB-839 administration inhibited the differentiation of monocytes to M2-polarized macrophages and suppressed the development of neovascularization after corneal alkali burn, which is consistent with previous reports in other models of ocular neovascularization. Therefore, the regulation of macrophage polarization may be a promising therapy for eye angiogenesis diseases.

Identification of the key molecules controlling M2 macrophage differentiation is the main challenge in interfering with angiogenesis. Recent metabolic studies revealed the crucial role of glutamine metabolism in shaping the polarization and functional plasticity of macrophages [15, 16–18, 42]. Several regulatory mechanisms for glutamine-mediated macrophage activation were proposed. Liu et al. reported that *α*-KG generated from glutaminolysis promoted the M2 phenotype via histone demethylase Jmjd3-mediated epigenetic reprogramming of M2-specific marker genes [18]. Jha et al. showed that UDP-GlcNAc was critical for M2 macrophage polarization because it was responsible for glycosylation of M2 marker proteins, and more than one-half of the nitrogen in UDP-GlcNAc was derived from glutamine [42]. We revealed that pharmacological inhibition and glutamine deprivation altered the biology of macrophages, which exhibited decreased cell proliferation and cell adhesion capacity and increased apoptosis of BMDMs. The number of intracorneal M2 macrophages was significantly reduced in CB-839-treated mice, but the number of M1 macrophages was not changed, which indicated that GLS1-mediated glutaminolysis facilitated M2 macrophage infiltration and activation and promoted macrophage secretion of proangiogenic cytokines, such as VEGF-A and PDGF-BB, to enhance experimental CoNV. M2 activation involves various transcription factors, including STAT3 and STAT6. Notably, our in vivo study also revealed that CB-8839 inhibited the activation of STAT3, but not STAT6. These findings are supported by a more recent study wherein the overexpression of miR-497 inhibited alkali burn-induced CoNV in mice by inhibiting macrophage infiltration and M2 polarization via targeting of the STAT3 signaling pathway [43]. Further studies are required to clarify the interaction mechanisms between glutamine metabolism and the STAT3 signaling pathway.

Previous studies identified that infiltrated monocytes and/or macrophages were a major source of VEGF-A and PDGF-BB in wound- and inflammation-related CoNV [44, 45]. VEGF-A has a proangiogenic effect on endothelial proliferation, migration, and capillary sprouting, and PDGF-BB promotes capillary maturation by recruiting pericytes to growing vessels [46, 47]. VEGF-stimulated endothelial cells produce high levels of PDGF-BB, which further aggravates vascular leakage [48]. Several research groups proposed that blockade of the VEGF-A and PDGF-BB pathways may be more effective for CoNV than blockade of either pathway alone [49, 50]. The present study revealed that treatment with CB-839 reduced IL4-induced VEGF-A and PDGF-BB mRNA and protein expression in BMDMs. These results are consistent with the reduction in CoNV by CB-839 treatment in vivo. CB-839-mediated inhibition of VEGF-A and PDGF-BB secretion by M2 macrophages was sufficient to suppress tube formation and the sprouting of endothelial cells, which indicates that these two factors are key mediators of angiogenesis.

CB-839 is a selective oral GLS1 inhibitor with improved potency and bioavailability in animal models compared to other small molecule glutaminase inhibitors, such as BPTES or DON [51, 52]. CB-839 is the only GLS inhibitor undergoing several different clinical studies. Therefore, the present study used CB-839, and its inhibitory effect was confirmed in metabolic assays. The method of administration and dose of CB-839 should be determined in future studies based on the physicochemical and pharmacokinetic properties.

## 5. Conclusion

Our data revealed a critical role of glutamine in maintaining the growth, adhesion, and polarization of macrophages. Our study identified the specific GLS1 inhibitor CB-839 as a potential therapeutic drug for the treatment of alkali-induced CoNV by inhibiting macrophage infiltration and M2 polarization. Overall, our results support GLS1 as a novel therapeutic target for CoNV.

## Data Availability

All data and materials are available upon request.

## Abbreviations

BMDM: Bone marrow-derived macrophage
CCL: C-C motif chemokine
CXCL: C-X-C motif chemokine ligand
CoNV: Corneal neovascularization
ELISA: Enzyme-linked immunosorbent assay
FGF2: Fibroblast growth factor 2
GLS1: Glutaminase 1
IL: Interleukin
IGF1: Insulin-like growth factor 1
ITGB2: Integrin beta 2
ICAM1: Intercellular adhesion molecule 1
LPS: Lipopolysaccharide
MRMEC: Mouse retinal microvascular endothelial cell
MMP9: Matrix metalloproteinase 9
OCR: Oxygen consumption rate
PDGF: Platelet-derived growth factor
PLGF: Placental growth factor
STAT3: Signal transducer and activator of transcription 3
TCA: Trichloroacetic acid
TLR: Toll-like receptor
VEGF-A: Vascular endothelial growth factor A.

## Supplementary Materials

Supplementary Materials Table S1: primers for real-time PCR detection. Table S2: antibodies for Western blotting in this study. Figure S1: glutamine (Gln) deprivation influences the basic biology of macrophages in vitro. Figure S2: targeting glutaminase 1 (GLS1) influences multiple metabolic functions in macrophages. Figure S3: glutamine (Gln) deprivation influences polarization of macrophages in vitro. Figure S4: targeting glutaminase 1 (GLS1) suppresses macrophage-mediated angiogenesis in vitro. Figure S5: histological assessment of corneal sections with H&E staining.
