# Supplementary material for: Pharmacological Inhibition of Glutaminase 1 Attenuates Alkali-Induced Corneal Neovascularization by Modulating Macrophages
Source: Oxid Med Cell Longev. 2022 Mar 19;2022:1106313. doi: 10.1155/2022/1106313 (PMC8957416; doi:10.1155/2022/1106313)
Supplement: Supplementary Materials — Table S1: primers for real-time PCR detection. Table S2: antibodies for Western blotting in this study. Figure S1: glutamine (Gln) deprivation influences the basic biology of macrophages in vitro. Figure S2: targeting glutaminase 1 (GLS1) influences multiple metabolic functions in macrophages. Figure S3: glutamine (Gln) deprivation influences polarization of macrophages in vitro. Figure S4: targeting glutaminase 1 (GLS1) suppresses macrophage-mediated angiogenesis in vitro. Figure S5: histological assessment of corneal sections with H&E staining. [file 1106313.f1.docx]

**Table S1. Primers for Real-time PCR detection**

| **Gene name** | **Accession number** | **Sequence (5′ to 3′)** |
| --- | --- | --- |
| IL-1β (Mouse) | NM_008361.4 | F: CACTACAGGCTCCGAGATGAACAAC  R: TGTCGTTGCTTGGTTCTCCTTGTAC |
| IL-6 (Mouse) | NM_001314054.1 | F: CTTCTTGGGACTGATGCTGGTGAC  R: TCTGTTGGGAGTGGTATCCTCTGTG |
| Mrc1 (Mouse) | NM_008625.2 | F: GTCTGAGTGTACGCAGTGGTTGG  R: TCTGATGATGGACTTCCTGGTAGCC |
| iNOS (Mouse) | NM_010927.4 | F: ATCTTGGAGCGAGTTGTGGATTGTC  R: TAGGTGAGGGCTTGGCTGAGTG |
| VEGFA (Mouse) | NM_001025250.3 | F: GGGCTCTTCTCGCTCCGTAGTAG  R: CCCTCTCCTCTTCCTTCTCTTCCTC |
| TNF-α (Mouse) | NM_001278601.1 | F: CGCTCTTCTGTCTACTGAACTTCGG  R: GTGGTTTGTGAGTGTGAGGGTCTG |
| CXCL11 (Mouse) | NM_019494.1 | F: GTAACGGCTGCGACAAAGTTGAAG  R: GAGGCGAGCTTGCTTGGATCTG |
| Chil3 (Mouse) | NM_009892.3 | F: GCCCACCAGGAAAGTACACAGATG  R: GACCTCAGTGGCTCCTTCATTCAG |
| Arg1 (Mouse) | NM_007482.3 | F: AGACAGCAGAGGAGGTGAAGAGTAC  R: AAGGTAGTCAGTCCCTGGCTTATGG |
| Retnla (Mouse) | NM_020509.4 | F: TCCCTCCACTGTAACGAAGACTCTC  R: CTCCCAAGATCCACAGGCAAAGC |
| IGF1 (Mouse) | NM_010512.5 | F: GCTCTGCTTGCTCACCTTCACC  R: CGGTCCACACACGAACTGAAGAG |
| PDGFB (Mouse) | NM_011057.4 | F: TCTCTGCTGCTACCTGCGTCTG  R: AGCCCCATCTTCATCTACGGAGTC |
| GLS1 (Mouse) | NM_001081081.2 | F: TGCGTTCCATGTTGGTCTTCCTG  R: TCCCTTAACACTGTTGCCCATCTTG |
| GLS2 (Mouse) | NM_001033264.3 | F: CATGCTGCCTCGACTTGGTGAC  R: GCCGTGGTGAACTTGTGGATAGG |
| IL-10 (Mouse) | NM_010548.2 | F: AGAGAAGCATGGCCCAGAAATCAAG  R: CTTCACCTGCTCCACTGCCTTG |
| CXCL10 (Mouse) | NM_021274.2 | F: TGCCGTCATTTTCTGCCTCATCC  R: TCCCTATGGCCCTCATTCTCACTG |
| TLR4 (Mouse) | NM_021297.3 | F: GAGCCGGAAGGTTATTGTGGTAGTG  R: AGGACAATGAAGATGATGCCAGAGC |
| MMP9 (Mouse) | NM_013599.5 | F: CGCCACCACAGCCAACTATGAC  R: CTGCTTGCCCAGGAAGACGAAG |
| TGF-β (Mouse) | NM_011577.2 | F: ACCGCAACAACGCCATCTATGAG  R: GGCACTGCTTCCCGAATGTCTG |
| CCL22 (Mouse) | NM_009137.2 | F: CTCCTGGTGGCTCTCGTCCTTC  R: TCCTGGCAGCAGATACTGTCTTCC |
| IL-12 (Mouse) | NM_001159424.2 | F: TCTTTGATGATGACCCTGTGCCTTG  R: GTGATTCTGAAGTGCTGCGTTGATG |
| PIGF (Mouse) | NM_008838.1 | F: GGCTGTGTATCTGCTCTGCTTCTG  R: GCTCGCGTTACCTTATGTGACAATG |
| FGF2 (Mouse) | NM_008006.2 | F: GAGCGACCCACACGTCAAACTAC  R: CAGCCGTCCATCTTCCTTCATAGC |
| PDGFA (Mouse) | NM_008808.4 | F: AAAGAGGTCCAGGTGAGGTTAGAGG  R: GTCTCCTCCTCCCGATGGTCTG |
| HGF (Mouse) | NM_001289458.1 | F: TGAGTTATGTGCTGGGGCTGAAAAG  R: ACCAGGAACAATGACACCAAGAACC |
| ACTIN (Mouse) | NM_007393.5 | F: TCCATCATGAAGTGTGACGT  R: TACTCCTGCTTGCTGATCCAC |

**Table S2. Antibodies for western blotting in this study**

| ***Name*** | ***Dilution concentration*** | ***Supplier*** | ***Lot No.*** |
| --- | --- | --- | --- |
| anti-Phospho-STAT1 | WB (1/1000) | Cell Signaling Technology | 9167 |
| anti-STAT1 | WB (1/1000) | Cell Signaling Technology | 9172 |
| anti- Phospho-STAT3 | WB (1/1000) | Cell Signaling Technology | 9145 |
| anti-STAT3 | WB (1/1000) | Cell Signaling Technology | 9139 |
| anti- Phospho-STAT6 | WB (1/1000) | Cell Signaling Technology | 56554 |
| anti-STAT6 | WB (1/1000) | Cell Signaling Technology | 5397 |
| anti- ICAM-1 | WB (1/1000) | Abcam | ab222736 |
| anti- ITGB2 | WB (1/1000) | Cell Signaling Technology | 72607 |
| anti- ITGAM | WB (1/2000) | Abcam | ab128797 |
| anti-GLS1 | WB (1/2000) | Proteintech | 66265-1-Ig |
| anti-GLS2 | WB (1/2000) | Abcam | ab113509 |
| anti-β-actin | WB (1/1000) | Cell Signaling Technology | 13E5 |
| Goat Anti-Mouse IgG H&L (HRP) | WB (1/5000) | Abcam | ab6789 |
| Goat Anti-Rabbit IgG H&L (HRP) | WB (1/5000) | Abcam | ab6721 |


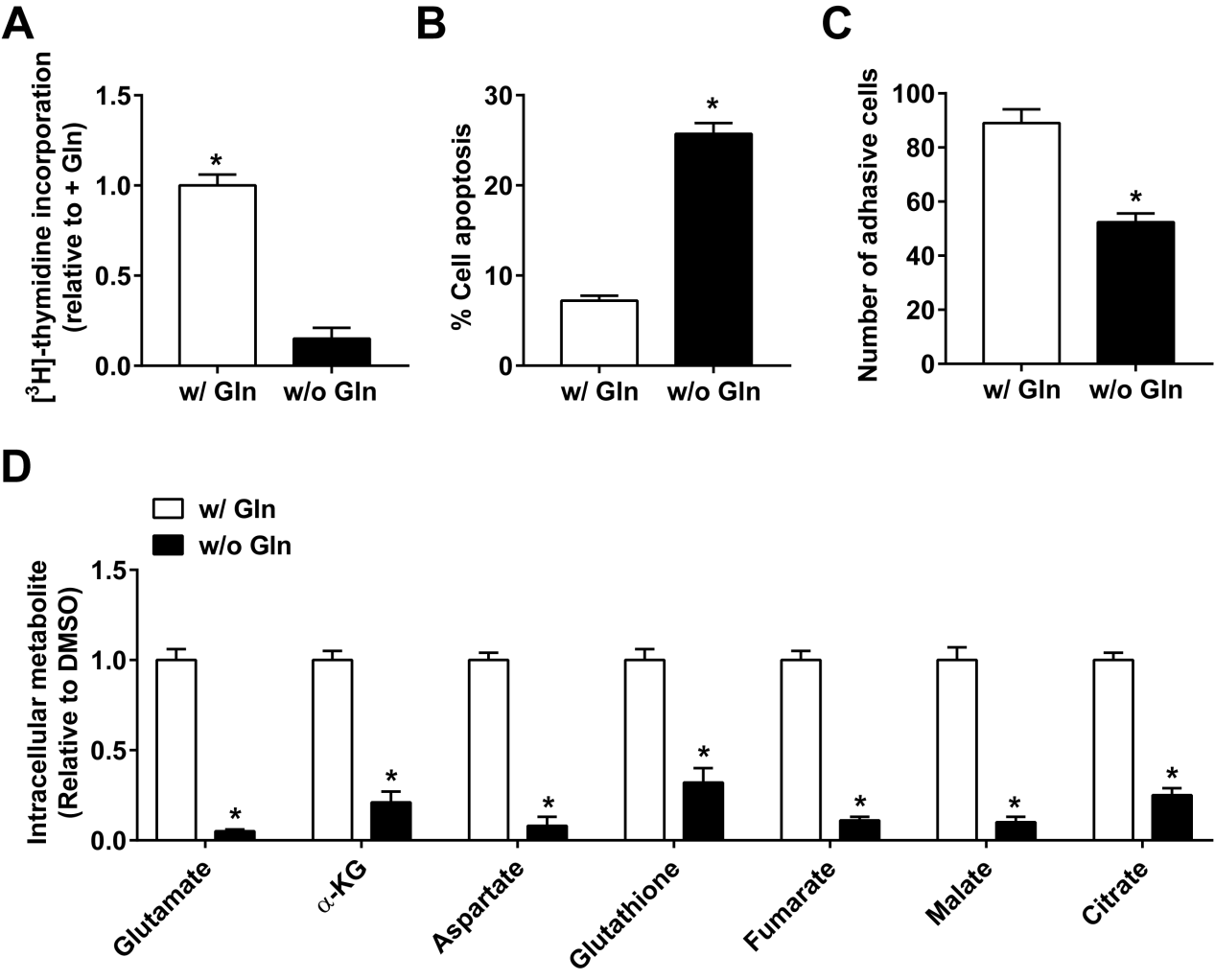


**Figure S1. Glutamine (Gln)** **deprivation influences the basic biology of macrophages in vitro**. **(A)** [^3^H]-Thymidine incorporation in DNA in BMDMs cultured in the presence (w/) and absence (w/o) of glutamine for 24 h. **(B)** Annexin-V/PI staining was performed to determine the percentage of apoptotic BMDMs. **(C)** Fluorescence microscope was applied to observe the number of adhesion of calcein-AM-labeled BMDMs to monolayers of MRMECs. **(D)** Quantification of intracellular TCA metabolites in control and glutamine-deprived BMDMs. Each experiment repeated three times and data in graphs represent means ± SEM. *P < 0.05


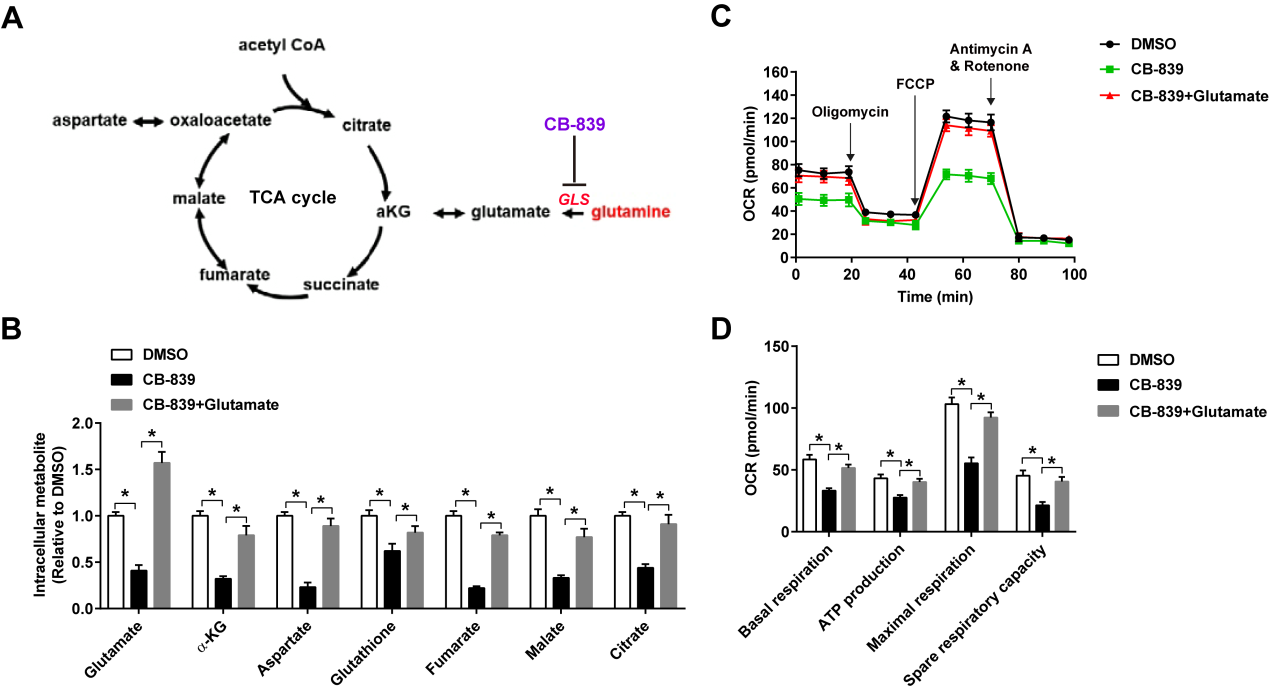


**Figure S2.** **Targeting glutaminase 1 (GLS1) influences multiple metabolic functions in macrophages** **(A)** Schematic of labeling scheme using [U-^13^C] glutamine. **(B)** Quantification of intracellular TCA metabolites in BMDMs treated with DMSO, CB-839 alone and CB-839 combined with glutamate supplementation. **(C)** Mitochondrial mito-stress assay in BMDMs after treatment by seahorse, cells were challenged with oligomycin (1 mM), FCCP (0.5 mM) and RO/AA (1 mM). **(D)** Quantification of basal respiration, ATP concentration, maximal respiration and spare respiration capacity in BMDMs. Each experiment repeated three times and data in graphs represent means ± SEM. *P < 0.05


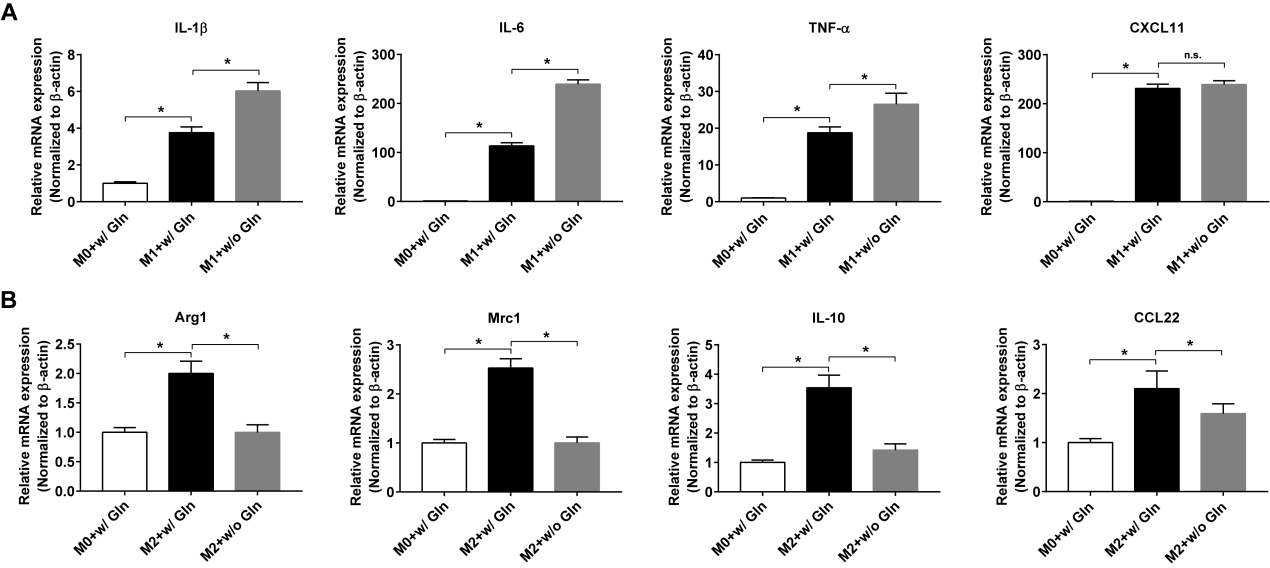


**Figure S3. Glutamine (Gln)** **deprivation influences polarization of macrophages in vitro.** RT-PCR analysis of M1 marker genes **(A)** and M2 marker genes **(B)** in uncommitted (M0) or LPS (M1) or IL-4 (M2) treated BMDMs under various culture conditions for 12 h. Each experiment repeated three times and data in graphs represent means ± SEM. ns, no significance, *P < 0.05

**
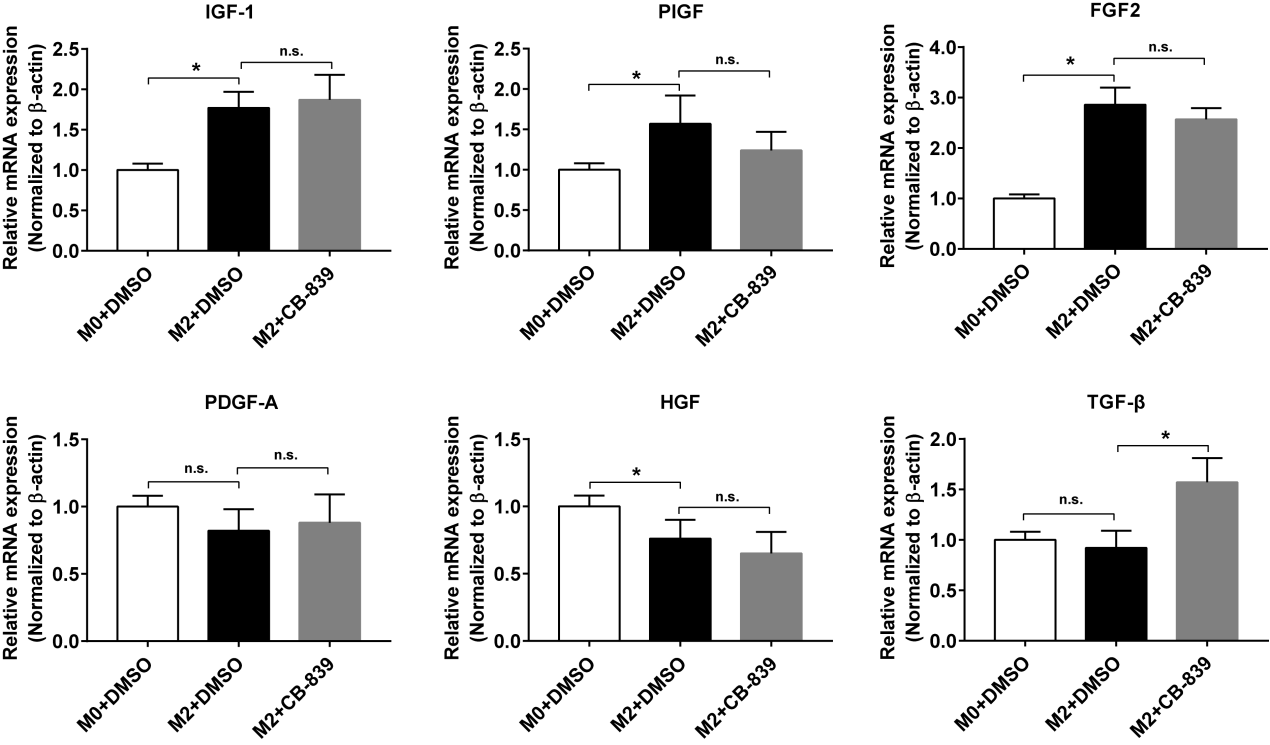
**

**Figure S4.** **Targeting glutaminase 1 (GLS1) suppresses macrophage-mediated angiogenesis in vitro.** RT-PCR assay were performed to determine the mRNA levels of proangiogenic factors in uncommitted (M0) or IL-4 (M2) treated BMDMs under various culture conditions for 12 h. Each experiment repeated three times and data in graphs represent means ± SEM. ns, no significance, *P < 0.05

**
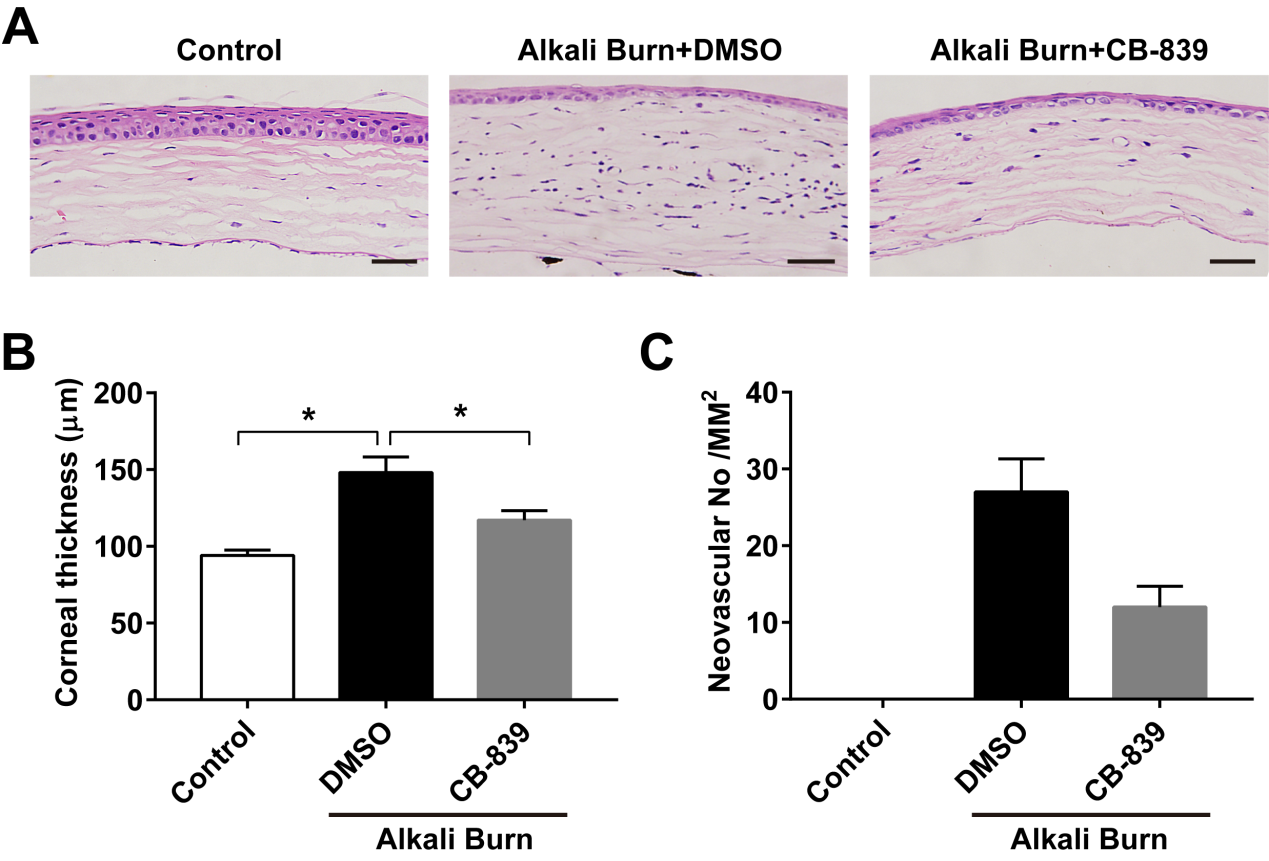
**

**Figure S5.** **Histological assessment of corneal sections with H&E staining. (A)** Representative H&E staining of the mouse corneas from different groups. Scale bars: 100 µm. **(B)** Corneal thickness were analyzed from corneas obtained from normal, or DMSO or CB-839-treated mice 7 days after injury. **(C)** CNV numbers per square millimeter in the “hot spots” (area of abundant neovascularization) were analyzed from normal, or DMSO or CB-839-treated mice 7 days after injury. n = 5-6/group for H&E staining. Each experiment repeated three times and data in graphs represent means ± SEM. *P < 0.05
